# Urinary Caffeine Levels in Chinese Children: Insights from Diet, Gender, and Regional Variations

**DOI:** 10.3390/nu17091594

**Published:** 2025-05-06

**Authors:** Wen-Jing Deng, John Chi-Kin Lee

**Affiliations:** 1 Department of Science and Environmental Studies, The Education University of Hong Kong, Tai Po, N.T., Hong Kong, China; 2 Academy of Applied Policy Studies and Education Futures, The Education University of Hong Kong, Tai Po, N.T., Hong Kong, China; jcklee@eduhk.hk

**Keywords:** urinary caffeine, Chinese children, dietary patterns, regional factors, EDI

## Abstract

Background/Objectives: The consumption of caffeine products among children in China is on the rise, yet there remains a paucity of evidence regarding the variability of caffeine consumption and the influencing factors. Urinary caffeine levels provide a valid indicator of caffeine intake, as it directly reflects the quantitative measure of consumption within the population. This study aimed to investigate the effects of diet, gender, and region on urinary caffeine concentrations in Chinese children and their parents, specifically focusing on participants from Guangdong province and Guangxi province. Methods: Morning urine samples were pretreated using liquid-liquid extraction. Caffeine and creatinine concentrations were analyzed using ultra-performance liquid chromatography coupled with triple quadrupole mass spectrometry (UPLC-MS/MS), with quality control measures ensuring method accuracy (recovery rate: 92.8–122%, RSD < 20%). Caffeine exposure was assessed through estimated daily intake (EDI) calculations, and statistical analyses, including weighted regression and Spearman correlation, were conducted to evaluate associations with demographic and dietary variables. Results: The findings revealed that urinary caffeine levels and detection rates among Chinese children were significantly lower than those observed in the United States (30.1 ng/mL), with a median concentration of 2.18 ng/mL and a detection rate of 45%. Urinary caffeine concentrations in males were slightly higher than in females; however, these gender differences were not statistically significant. Certain dietary habits, particularly milk consumption, were found to influence urinary caffeine levels and detection rates. Using the random forest method, urine caffeine detection was highest (nearly 60%) when milk was consumed three times a week. Regionally, children in Guangdong had marginally higher urinary caffeine concentrations compared to those in Guangxi (median: 5.20 μg/g_crea_ vs. 1.58 μg/g_crea_). The estimated daily caffeine intake suggested that children in China consume less caffeine than their counterparts in other countries. Conclusions: These results indicate that dietary habits significantly correlated with caffeine consumption in children, and urinary caffeine concentration serves as a valuable measure for dietary research.

## 1. Introduction

According to our preliminary study conducted in 2021–2022, we found caffeine in the urine of children in Hong Kong and Guangzhou, which warrants particular concern [1] Caffeine (1,3,7-trimethylxanthine) is the most widely consumed psychoactive substance, present in numerous products, including food, beverages, and medications, and it is an integral component of contemporary diets [2]. The pharmacokinetics of caffeine clearance in children and adolescents is comparable to that of adults, and habitual caffeine consumption is generally considered not to have an adverse effect on the health of young individuals. However, recent studies suggest that caffeine intake in children and adolescents should be monitored and potentially restricted [3,4,5]. The daily consumption of energy drinks containing caffeine has been significantly associated with adverse effects, including headaches, sleep disturbances, and fatigue [6]. Children are less likely to experience the benefits of regular caffeine consumption compared to adults and are more susceptible to withdrawal symptoms [7]. Caffeine overdose can lead to tachycardia, tremors, hypertension, irritability, impulsive behavior, and, in severe cases, sudden death [8]. Additionally, caffeine may adversely affect the nervous and cardiovascular systems in adolescents, with associations reported for anxiety, tension, migraines, gastrointestinal disorders, metabolic acidosis, insomnia, arrhythmias, and other cardiovascular issues. Such neurological and physiological effects are particularly concerning for children due to their developmental immaturity [4,9]. In response to these concerns, the European Food Safety Authority (EFSA) and the Canadian Health Department have established daily caffeine intake limits for school-age children and adolescents, a policy that has also been adopted in China [10,11].

As the global population continues to grow and cross-border trade becomes more prevalent, total caffeine intake has increased, correlating strongly with gross domestic product [12]. These shifts have influenced the dietary culture of several nations, resulting in higher caffeine consumption among children and adolescents, particularly in certain regions. For example, young individuals in Italy, aged 12 to 19 years, consume an average of 125.2 mg of caffeine per day [13]. Similarly, in China, rising GDP, increasing international trade, and greater consumption of coffee and caffeine-containing beverages among children and adolescents have been noted, alongside dietary changes involving caffeine-rich products [14]. Tong et al. [15] reported that the average daily caffeine intake among Chinese children and adolescents increased from 0.12 mg/kg body weight per day in 2004 to 0.30 mg/kg body weight per day in 2024, with the proportion of caffeine consumers rising from 4.9% to 27%. Moreover, carbonated beverages have overtaken traditional tea as the primary source of caffeine for this demographic, accompanied by increasing consumption of energy drinks and coffee [16]. Despite this, the majority of research on caffeine consumption in China has been conducted at the provincial or city level or has focused on broad regional comparisons, such as southern versus eastern China. These studies often emphasize direct dietary factors associated with caffeine without considering the influence of overall dietary pattern modifications, such as changes in meat or seafood intake, on caffeine consumption. Furthermore, less attention has been paid to other contributing factors affecting caffeine intake, such as family dietary habits, urbanization, and intergroup population differences.

Most studies evaluating caffeine consumption among children have relied on dietary questionnaires. However, these tools have inherent limitations, including inadequacies in self-reporting, particularly among adolescents, and challenges in accurately identifying all sources of caffeine [17]. Self-reported data can be influenced by recall biases, misclassification, and other measurement inaccuracies, particularly in studies involving children [16]. In contrast, caffeine and its metabolites in urine have been identified as valid biomarkers for caffeine consumption [18,19]. Approximately 1–2% of ingested caffeine is directly excreted in urine, while the remainder is metabolized into theobromine, paraxanthine, and other metabolites [20]. Studies have demonstrated a positive correlation between urinary caffeine levels and self-reported 24-h caffeine intake, highlighting the utility of urinary analysis as an objective measurement tool [18]. In comparative studies involving urinary caffeine and its metabolites correlated with questionnaire-based caffeine intake, urinary caffeine outperformed other metabolites [19].

Over 250 urine samples were collected in Guangzhou, Guangdong Province, and Hechi, Guangxi Province, for caffeine testing. In conjunction with urine sample collection, a comprehensive questionnaire survey was conducted. Caffeine concentrations in urine samples were determined for children from the Guangdong and Guangxi provinces of China, which share similarities in dietary and geographical conditions. Further analysis was performed on dietary habits, gender, and regional differences that may influence urinary caffeine levels in Chinese children. The aim of this study is to investigate the urinary caffeine levels in children from Guangdong and Guangxi provinces of China, exploring the relationship between caffeine consumption and various influencing factors, including dietary habits, gender, and regional differences. This research seeks to provide objective insights into caffeine intake among children, addressing potential health concerns associated with increased consumption of caffeinated products in contemporary diets.

## 2. Materials and Methods

### 2.1. Urine Collection/Pretreatment, Chemicals and Materials

Morning urine samples were collected from 77 parent-child pairs (97 children and 116 parents) in Hechi, Guangxi and 40 children in Guangzhou, Guangdong from July to August 2023. A total of 137 children (aged 3–13 years), with 57% of whom being female, and 116 volunteer parents participated in the study. Additional details of the participants, including age, gender, height, weight, body mass index, and obesity rates, are provided in Table S1. Collected urine samples were immediately frozen at −20 °C and transported to the laboratory in pre-cleaned glass containers. The urine pretreatment method was conducted using liquid-liquid extraction (see Supplementary Document for further details). Creatinine-corrected concentrations (µg/g creatinine) or (µg/g_crea_) were utilized in this study.

Methanol, ethyl acetate, ammonium formate, acetonitrile, caffeine, creatinine, and creatinine-d3 were purchased from Merck (Steinheim, Germany). Methyl tert-butyl ether and formic acid were obtained from CNW Technologies GmbH (Düsseldorf, Germany). Milli-Q water was produced using a Milli-Q integrated water purification system (Stockholm, Sweden). The enzyme reagent of β-Glucuronidase (>100,000 units/mL)/Arylsulfatase (<20,000 units/mL) was purchased from Sigma-Aldrich Company (St. Louis, MO, USA). All chemicals had a purity of at least 98%.

The pretreated samples were analysed for caffeine and creatinine using external standard methods. Analysis was conducted using a LC system coupled with an Xevo TQ-S triple quadrupole mass spectrometer (Waters, Milford, MA, USA).

### 2.2. Quality Control

To ensure the accuracy of the analytical method, the reliability of the instruments, and the absence of contamination in experimental materials, method recovery assessments for the target substances were conducted prior to sample analysis. A solvent blank, procedural blank, and quality control sample (containing a mixture of 50 ppb targets) were included for every ten urine sample analyses. The solvent blank, prepared with methanol, confirmed the absence of residues in the instrument during analysis; the procedural blank, substituted with pure water, verified that no external target substances were introduced during the analysis. Quality control samples were used to monitor instrument stability throughout the analysis. The caffeine concentration in all blank samples was below the detection limit, and the relative standard deviation (RSD) for all quality control samples was less than 20%. The recovery rate of target substances in urine samples ranged from 92.8% to 122%. The limits of detection (LOD) and quantification (LOQ) were defined as 10 times and 3 times the signal-to-noise ratio, respectively. Table S2 provides detailed information on mass conversion factors and retention times for caffeine and creatinine during the analysis process.

### 2.3. Health Risk Assessment

The estimation of daily intake (EDI) and health risk assessment for caffeine was conducted using unadjusted data. Currently, most studies estimate EDI using questionnaire-based methods, and no precise formula exists to directly assess caffeine intake-related risks based on urinary caffeine concentrations. However, previous studies have demonstrated a significant correlation between urinary caffeine concentrations and caffeine intake reported in questionnaires [18]. Therefore, EDI was estimated using the metabolic formula for other pollutants in urine as follows: EDI = (C × V)/(F × Bw), where C represents the concentration of caffeine in urine (μg/mL), V is the average daily urine output (mL/kg/d), F is the excretion fraction, and Bw is the body weight of the children. Based on a meta-analysis, the estimated 24-h urine volume for children is approximately 461 mL/24 h for those aged 2–5 years, 758 mL/24 h for children aged 6–12 years, and 1048 mL/24 h for adolescents aged 13–19 years [21]. The comprehensive excretion fraction of caffeine is approximately 1.2% [22].

### 2.4. Statistical Analysis

Urinary caffeine concentrations were adjusted for urinary creatinine, and these adjusted concentrations were used to compare and analyze variations across different populations and their responses to questionnaire variables. The questionnaire variables included gender, height, weight, age, frequency of egg consumption, frequency of fried food consumption, frequency of seafood consumption, frequency of chicken, duck, beef, and lamb consumption, frequency of packaged food consumption, and frequency of milk consumption. The variables were summarized using geometric means and interquartile ranges. Weighted regression analysis was performed on different variables using R (2023 version). Spearman correlation analysis, as well as *t*-tests or Mann-Whitney U tests for differences in the same variable across regions, populations, or genders, were conducted using Python (2023 version). Random forest analysis was applied to variables that exhibited significant differences to predict their specific impact on caffeine concentration.

## 3. Results and Discussion

### 3.1. Urinary Caffeine Concentration and Creatinine-Adjusted Caffeine Concentration

**Figure 1:**
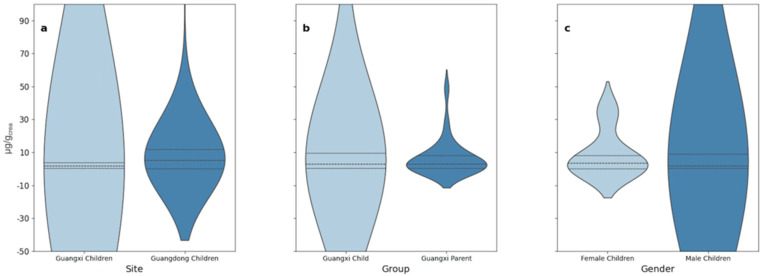
A violin plot comparing caffeine levels (μg/g_crea_) between different sites (Guangxi and Guangdong) (**a**); (**b**) groups (children and parents of Guangxi), and (**c**) genders (male and female children).

This section displays the concentrations of urinary caffeine (μg/g_crea_) across the groups after controlling creatinine levels using standard methods [23] (Figure 1, Table 1). The unadjusted urinary caffeine data (ng/mL) are presented in Table S3.

The adjustment for creatinine levels was performed for several key reasons. Firstly, meta-analyses and clinical studies indicate that caffeine does not affect the glomerular filtration rate or influence creatinine metabolism [24]. Secondly, this adjustment mitigates the significant fluctuations in urinary caffeine data that arise from variations in urine flow rates and concentrations of other substances, thereby enhancing data credibility [25]. Moreover, caffeine’s diuretic effect can lead to increased urine output [26], which dilutes urinary caffeine concentrations and introduces inaccuracies in unadjusted data. This issue is further compounded by individual differences in water consumption, such as higher water intake among overweight individuals [27], which can amplify the variability of first-morning urine samples. Thus, creatinine adjustment is essential to account for these factors and ensure the reliability of the results.

### 3.2. Urine Caffeine in Children

The detection rates and urinary caffeine concentrations documented in this study were significantly lower than those reported in comparable studies from other countries, especially the United States and European countries. For example, a survey conducted in the United States in 2010–2011, the detection rate of caffeine was 90.8% of persons aged 6 years and older, and the median concentration of caffeine in the urine of children aged 2–11 years was 30.1 ng/mL [28]. Likewise, in Switzerland, the detection rate for children aged 6–16 years was 98.14%, with a median urinary caffeine level of 200 ng/mL [19]. In older groups, such as young adults aged 18–30 years in Canada, the median urinary caffeine concentration was even higher at 822 ng/mL [29]. In contrast, the highest detection rate in our study was 45% in the Guangdong region, while the median urinary caffeine concentration in the Guangxi region was only 2.180 ng/mL. These differences are likely due to dietary habits, cultural factors, and living environments. In the United States, caffeine consumption among children is more diverse, with primary sources including chocolate products, soda, energy drinks, tea, flavored dairy products (for children under 12 years), and coffee (for those aged 12 years and older) [30]. Studies have shown that even in the youngest age group (2–5 years old), the proportion of caffeine product consumers reaches 43% [31]. This high consumption rate may be partly attributed to more lenient parental dietary management and greater opportunities for children to consume fast food rather than meals at home or in cafeterias, as fast-food restaurants are a common source of soda purchases [7].

**Figure 2:**
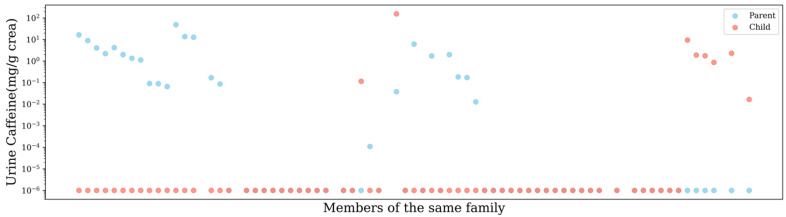
Dots of caffeine intake for parents and children in each Guangxi family.

On the other hand, in China, 80% of adolescents in the age group 11–17 years consume foods and beverages from home or school canteens, which are essentially free of caffeine [32,33]. Exploratory analysis of urinary caffeine levels across parents and children within families showed that 55.1% of the families had urinary caffeine concentrations in all households below the LOD (Figure 2). This result supports the probability that shared family meals are primarily non-caffeinated meals and snacks. Furthermore, raising parental concern about the effects of health implications arising from the consumption of sugary products such as drinks and chocolates has seen the number of restrictions imposed on children’s consumption of these products increase [34]. As a result, most Chinese children and adolescents drink cola, tea drinks, and energy drinks with caffeine less than once per week, while their consumption of candies and pastries is also relatively low [35]. As mentioned earlier, the consumption of caffeine-containing products in China is already on the rise, but it is still much lower than that of Western countries. According to the National Food and Beverage Consumption Survey in 2018, only 18.3% of Chinese adolescents consumed caffeine, and the main dietary sources were traditional tea, soda, and tea products [15]. Although previous studies in China may have overlooked certain caffeine-containing foods, such as chocolate milk, potentially leading to an underestimation of the data, we still believe that the proportion of caffeine consumers and the daily caffeine intake among Chinese adolescents and children are significantly lower than those in Western nations.

In order to understand the correlation of urinary caffeine concentrations with BMI, weight, gender, region, and parent-child, Spearman correlation tests and Mann-Whitney U tests were conducted. Overall, no significant correlations or differences were observed. However, if we look at the data from families, we can see that in only 11.6% of parent-child dyads, children had higher levels of caffeine in their urine than their parents. Except for families where neither parents nor children had detectable caffeine levels, parental caffeine excretion was higher than that of children in most families, as depicted in Figure 2. We performed a Wilcoxon signed-rank test to analyze caffeine intake in parent-child pairs (averaging values for multiple children or parents in the same family) and found a significant difference (Statistic = 134.0, *p* = 0.025). This has a connotation that children have limited access to caffeine-containing foods or beverages and are less inclined to consume such products. This trend is consistent with findings from studies conducted in China and other regions of the world [15,36].

### 3.3. Regional Difference

In the present study, we targeted sampling areas in South China, including Guangdong and Guangxi. According to the national caffeine consumer survey, the rate of caffeine users in South China, including these two regions, is 15.2% [15]. This rate is slightly higher than the urinary caffeine detection rate in Guangxi (13.4%) but significantly lower than that in Guangdong (45%). In addition, the median creatinine-adjusted caffeine level in Guangdong was significantly higher than that in Guangxi at 5.20 μg/g_crea_ compared to 1.58 μg/g_crea_. Based on these results, caffeine intake and the percentage of caffeine consumers are also higher in Guangdong than in Guangxi.

One possible explanation is that the Guangdong region has stronger economic development and international trade activities, which introduce more caffeine products from domestic and international such as energy drinks, soft drinks, and chocolate products to the market. This competitive environment likely enhances the advertising and promotional efforts for caffeine-containing products in the Guangdong region. Numerous studies have corroborated the impact of advertising campaigns, peer recommendations, and social elements on the consumption of energy drinks [37].

### 3.4. Age Difference

The age range of participants was initially 3–13 years. However, after excluding data below detection limits, the adjusted age ranges were 3–10 years for Guangxi and 3–8 years for Guangdong. In Guangxi, the average caffeine levels were found to be 2.59 μg/g_crea_ for children aged 3–6 years and 3.46 μg/g_crea_ for those aged 7–10 years, indicating that older children had higher caffeine levels. In Guangdong, the average caffeine level across these ages was 7.76 μg/g_crea_, with the highest level observed in 5-year-olds, averaging 26.8 μg/g crea, who are among the oldest in kindergarten.

### 3.5. Eating Habits

**Figure 3:**
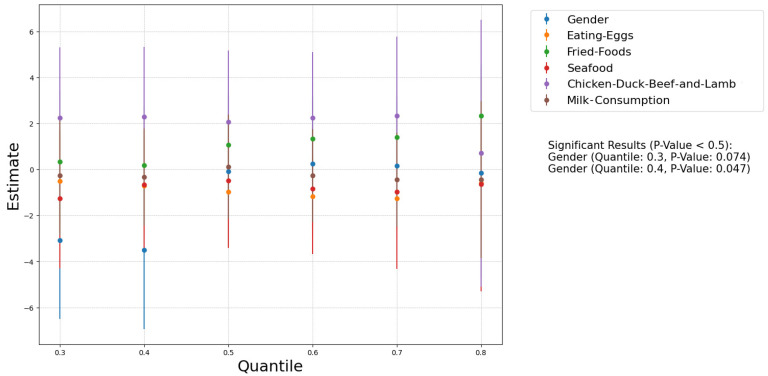
Quantile regression of dietary habits (without direct caffeine intake) and body weight: influence of gender and food consumption over quantiles.

**Figure 4:**
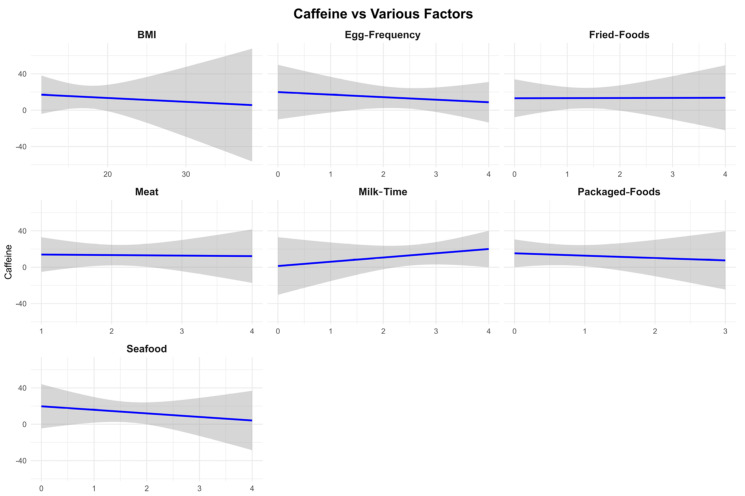
Regression models compared urine caffeine with other factors.

We used Spearman correlation tests and quantile regression analyses (Excluding values below the detection limit) to explore the associations of dietary patterns with creatinine-adjusted urinary caffeine levels (Figure 3). Among the dietary factors assessed, only the frequency of dairy consumption exhibited a significant correlation with urinary caffeine levels (Spearman ρ: 0.26, *p* = 0.0076). Due to the data distribution issues, we performed weighted regression analysis to create the Faceted Linear Regression Plot and obtained the same results (Figure 4).

Although seafood and egg consumption did not significantly correlate with urinary caffeine concentrations, both were negatively associated with caffeine levels. Eggs are well-known for their positive impact on longevity, health, and lifestyle, suggesting that egg consumption is part of a healthy diet [38]. While other studies have linked high egg intake to negative health impacts [39], the frequencies of intake in this study were considerably lower, allowing eggs to be viewed as an indicator of better nutrition within this sample. On the other hand, poor dietary habits are more likely to expose individuals to caffeine-containing foods or drinks. Caffeine intake is, however, often linked to high-calorie foods, beverages and chocolate products, among other foods and beverages. These poor dietary habits are also likely to cause increased contact with caffeine-containing products.

Seafood and meat consumption have been shown no direct impact on tea or coffee intake [40]. The consumption of seafood in China is more from home than from fast-food joints, which may have lowered the indirect contact with caffeine products. Nevertheless, more research is required to support this hypothesis, especially with regard to the origin of seafood consumed.

**Figure 5:**
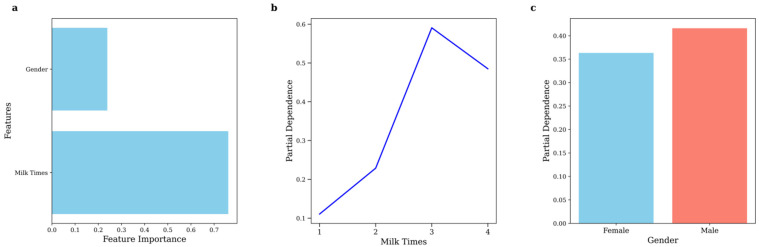
(**a**) Feature importance (gender and frequency of drinking milk), (**b**) Partial dependence of frequency of drinking milk, (**c**) Partial dependence of frequency of gender.

Since gender, dairy consumption frequency, and urinary caffeine levels were significantly different between male and female participants, we used a random forest classification model to assess the combined impact of the variables (Figure 5). The model yielded an accuracy of 0.69, and the model was used to determine the probability of caffeine presence in urine. The findings showed that the chance of detecting caffeine was higher for people who consumed dairy and reached the highest point at 60% for people who consumed three servings of dairy per day. But this likelihood was only slightly lowered when consumption reached four portions per day. The breakdown of this picture shows that this trend is highly correlated with flavoured milk. Research on adolescents in the United States of America has revealed that about one-fifth of the total dairy intake by children between the ages of 2–5 years is from flavoured milk, with a slight rise before the age of 11 years [41]. Likewise, caffeinated chocolate and cocoa milk products are popular in both China and the United States [42,43], implying that higher milk consumption may increase the likelihood of consuming caffeinated dairy products. This finding is in concordance with a cross-sectional study of NHANES data on caffeine consumption among adolescents in the US, which pointed to flavoured dairy as the main source of caffeine for children under 12 years [30].

However, most extant research on caffeine consumption among Chinese children and adolescents has failed to consider this source, which may have led to underestimation [15]. These data point to the relative importance of dairy products in determining urinary caffeine levels. Further research in this area should incorporate a finer categorization of dairy types, so as to determine the impact of caffeinated dairy products on overall caffeine consumption in dietary surveys.

### 3.6. Gender Difference

Our data did not show a significant correlation between gender and urinary caffeine concentrations. Nevertheless, using quantile regression analysis, a slight but notable correlation was observed, but very weak and significant only at the 30th (*p*-value: 0.074) and 40th (*p*-value: 0.047) percentile (Figure 3). Among female children, 81.6% were below the 30th percentile of urinary caffeine level, while among male children, it was 75%. Notably, two outliers with exceptionally high urinary caffeine concentrations were both males: one boy from Guangdong (152.6 μg/g_crea_) and another boy from Guangxi (462.2 μg/g_crea_). In addition, the caffeine detection rate in females (19.3%) was significantly lower than in males (28.0%); this indicates that girls have a significantly lower tendency to use or be exposed to caffeine products compared to boys. Based on the results of the percentile correlation analysis, males had a higher proportion in the lower percentiles, suggesting that males are more likely to consume caffeine products as part of their daily diet or beverages. However, no significant gender differences were observed in the other percentiles. These outcomes were further supported by the random forest model, showing a greater probability of caffeine consumption among males, albeit not a significant one (Figure 5).

The findings might be attributed to the fact that energy drinks are commonly consumed, and there are gender differences in the acceptance of caffeine products. Several authors have found that Males had a higher tendency to consume energy drinks than females [37,44]. Alabbad et al. [37] have attributed this behaviour to male characteristics like taking risks, being more physically active, being less attentive, and delaying academic or personal work. Hoyte et al. also showed that over 70% of college athletes use sports drinks to enhance performance in sport, with males still significantly outnumbering females in this group [45].

Gender differences in the physiology and psychology of humans can also affect the amounts of caffeine taken. It has also been established that males have a propensity to record enhanced, rewarding, stimulating, joyful, and even pleasurable effects of caffeine. The females, probably due to the action of steroid hormones like estradiol, claim less positive or even negative effects of caffeine [46]. This sensitivity may make females more susceptible to the adverse effects of caffeine, such as impulsive or risky behaviours like drinking or fighting [8]. Consequently, females may be less inclined to consume caffeine-containing products, particularly at higher intake levels. These differences help explain why caffeine consumption and the highest caffeine intake groups (outliers) are predominantly male in both this study and others.

Notably, while 74% of the male participants tested positive for caffeine, a higher proportion of participants with higher urinary caffeine levels were male, and the median urinary caffeine concentration was higher among females. From observing the data structure, we found that in the overall percentile distribution of urinary caffeine, male data is more concentrated in the lower and higher percentiles, while in the middle percentiles, it is slightly less than that of females. This outcome is influenced not only by the limitations of our sample collection timing but also by the inherent limitations and impacts of using urinary caffeine concentrations as a substitute for caffeine intake data. Using urinary caffeine concentrations as a measure of intake will produce underestimation for males and overestimation for females. This difference might be explained by physical activity, which increases the rate of caffeine metabolism and raises secondary metabolites, including paraxanthine and theobromine [47]. Structured and vigorous physical activity is more common among boys than girls [48]; therefore, they may decrease urinary caffeine levels even more. On the other hand, changes in caffeine clearance during the female menstrual cycle were reported; for instance, reduced clearance in the late luteal phase might be involved in variation; however, this is considered to be not of clinical significance [49].

### 3.7. EDI Estimation of Caffeine

**Figure 6:**
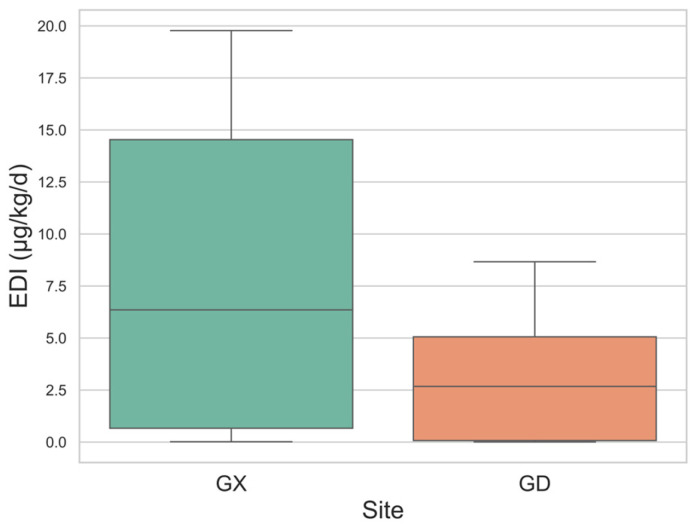
EDI (Estimated Daily Intake) distribution of children (GX—Guangxi, GD—Guangdong).

Due to incomplete height and weight information for parents, we estimated the predicted EDIs (Table S4 and Figure 6) based solely on urinary caffeine concentrations in children. The EDI data indicate that children from the Guangxi region had a significantly higher average EDI (7.666 μg/kg/day) and median EDI (6.354 μg/kg/day) compared to children from the Guangdong region, whose average EDI was 2.941 μg/kg/day and median EDI was 2.676 μg/kg/day (Table S4 and Figure 6).

These EDIs were significantly lower than those reported in other countries, including Canada (median 60 µg/kg/day for ages 2–3), the United States (Median 110 µg/kg/day for ages 2–5), and Switzerland (Median 300 µg/kg/day for ages 6–16) [19,31]. Additionally, they fall well below the intake limits recommended by the European Food Safety Authority (EFSA), which advises a maximum of 3 mg/kg/day for children, and Health Canada, which recommends a limit of 2.5 mg/kg/day for those under 12 [11,50].

These results suggest that caffeine consumption among children in both Guangxi and Guangdong is moderate and likely not hazardous to their health. However, it is important to note that our estimates may underestimate actual caffeine intake. Caffeine is primarily metabolized by the CYP1A2 enzyme into paraxanthine and other metabolites, with only about 1.2% (ranging from 0.5% to 2%) of ingested caffeine excreted unchanged in urine over 48 h [20,22]. Petrovic et al. found a strong correlation between caffeine consumption from coffee and urinary caffeine concentration, noting that paraxanthine levels could differentiate among various consumer groups [18].

In conclusion, while caffeine consumption among children in Guangxi and Guangdong appears to be within safe levels, potential underestimations warrant caution. Future studies should consider using EDI estimates derived from metabolites, such as paraxanthine, which may provide a more accurate assessment of caffeine exposure.

## 4. Conclusions

The findings of this study indicate that caffeine consumption among children in Guangdong and Guangxi, China, is on the rise but remains lower than levels reported in many other countries. Increased domestic consumption and international trade may be driving this growth, influenced by advertising and new product introductions. However, many dietary habits appear to have minimal negative impacts on caffeine intake, possibly due to the nature of consumed foods, such as seafood, which align with healthier lifestyles. Additionally, a positive correlation exists between milk consumption and caffeine intake, likely due to chocolate milk, an aspect often overlooked in caffeine studies in China.

Our results suggest that urinary caffeine levels could serve as a reliable biomarker for assessing caffeine intake, potentially replacing self-reported questionnaires, though further method development is needed. Future research should explore the relationship between urinary caffeine metabolites and consumption patterns, as well as the feasibility of 24-h urine sample collection to enhance data reliability. Expanding the sample size and including guardians in the analysis could improve insights into children’s caffeine exposure and dietary habits. Despite current caffeine intake being below recommended levels, ongoing longitudinal studies are essential to understand the factors influencing caffeine consumption in Chinese children and its potential health implications. Addressing these research gaps will deepen our understanding of caffeine intake patterns and their effects.

## Figures and Tables

**Table 1:** Caffeine in Urine excretion statistics.

	Sample Size	Mean	Median	25th Percentile	75th Percentile	Range
Guangdong-Children (µg/g_crea_)	40	48.26	5.201	0.053	11.72	0.00615–152.6
Guangxi-Children (μg/g_crea_)	97	26.35	1.581	0.228	4.013	0.0164–462.2
Guangxi-Parent (μg/g_crea_)	116	8.304	4.304	0.533	8.737	0.0655–48.74
Children in total (μg/g_crea_)	137	26.35	3.400	0.108	9.423	0.00615–462.2

## Data Availability

The authors confirm that the data supporting the findings of this study are available within the article and its Supplementary Materials. Raw data that support the findings of this study are available from the corresponding author upon reasonable request.

## Supplementary Materials

The following supporting information can be downloaded at: https://www.mdpi.com/article/10.3390/nu17091594/s1, Table S1: Characteristics of the participants; Table S2: Mass Transitions and Retention Times for Caffeine and Creatinine Analyzed by HPLC-MS/MS Using the Waters XEVO TQ-S System; Table S3: Caffeine in Urine excretion statistics; Table S4: Estimated daily intake (EDI) of caffeine predicted by urine.
