# Supplementary material for: Urinary Caffeine Levels in Chinese Children: Insights from Diet, Gender, and Regional Variations
Source: Nutrients. 2025 May 6;17(9):1594. doi: 10.3390/nu17091594 (PMC12073752; doi:10.3390/nu17091594)
Supplement: Supplementary file 1 [file nutrients-17-01594-s001.zip › nutrients-3591967-supplementary.pdf]

Assessing Urinary Caffeine Levels and Influencing Factors in Chinese Children

Wen-Jing Deng[a*], John Chi-Kin Lee[b]

[a] *Department of Science and Environmental Studies, The Education University of Hong Kong, Tai Po, N.T., Hong Kong, China*

[b] *Academy of Applied Policy Studies and Education Futures, The Education University of Hong Kong, Tai Po, N.T., Hong Kong, China*

Table S1: Characteristics of the participants

		GD-Children	GX-Children	GX-Parent
Total Population		40	97	116
Valid data quantity		16 (45%)	13 (13.4%)	25(21.55%)
Age (years)	Mean	5.5	5.9	-
	range	3-8	3-13	-
Male (%)		56%	57%	55%
Height (cm)	Mean	114.2	113.3	-
	range	100-130	94-130	-
Weight (kg)	Mean	23.16	19.03	-
	range	18-34	15.5-24	-
Body mass index (kg/m^2)	Mean	17.82	14.84	-
	range	12.43-27.60	13.14-16.53	-
Overweight (%)		43.70%	10%	-

Table S2: Mass Transitions and Retention Times for Caffeine and Creatinine Analyzed by HPLC-MS/MS Using the Waters XEVO TQ-S System

Compound	Parent (m/z)	Daughter ion 1 (m/z) and collision energy (eV)	Daughter ion 2 (m/z) and collision energy (eV)	Retention Window (min)
Caffeine	195.1	42.1 0(40)	138.1 (16)	3.5
Creatine	114.0	43.97 (13)	86.05 (11)	3.5
Creatine-d3	117.0	47.03 (13)	89.07 (11)	3.5

Table S3: Caffeine in Urine excretion statistics

	Geometric mean	Mean	Median	25th percentile	75th percentile	Range
Hechi-Children (ng/mL)	0.749	2.723	2.180	0.348	5.275	0.0083-6.4
Hechi-Children (μg/g_{crea})	1.35	48.263	1.581	0.228	4.013	0.0164-462.181
Guangzhou-Children (ng/mL)	0.276	1.493	1.439	0.029	2.763	0.0038-3.5147
Guangzhou-Children (μg/g_{crea})	1.11	17.292	5.201	0.053	11.716	0.00615-152.57
Children (ng/mL)	0.423	2.020	1.439	0.040	3.502	0.0038-6.4
Children (μg/g_{crea})	1.19	26.345	3.400	0.108	9.423	0.00615-462.181
Hechi-Parent (ng/mL)	1.017	3.200	3.208	0.260	6.325	0.0002-6.816
Hechi-Parent (μg/g_{crea})	2.39	8.304	4.304	0.533	8.737	0.0655-48.737

*Corresponding author. wdeng@eduhk.hk (Dr. Wen-Jing Deng)

Table S4: Estimated daily intake (EDI) of caffeine predicted by urine

	Geometric mean	Mean	Median	25th percentile	75th percentile	Range
Guangzhou-Children (μg/kg/d)	0.581	2.941	2.676	0.075	5.059	0.00554-8.667
Hechi-Children (μg/kg/d)	1.958	7.666	6.354	0.666	14.535	0.0159-19.778
Children in total(μg/kg/d)	0.978	4.966	2.676	0.102	7.497	0.00554-19.778

S1: Determination of with Caffeine and Creatinine

Urine samples were sent to the laboratory and stored in a freezer at -20°C. Before starting the urine pretreatment process, the samples were first thawed at room temperature. After thawing, the samples were stirred for 30 seconds. Then, 1 mL of urine was transferred into a 15 mL glass centrifuge tube. Subsequently, 0.5 mL of ammonium acetate buffer solution (7.71 g ammonium acetate, 93.8 mL MQ water, 6 mL acetic acid, and 200 μL β-glucuronidase), 100 ppb internal standard, and 1 mL of MQ water were added to the centrifuge tube in sequence and mixed thoroughly. After mixing, the samples underwent enzymatic hydrolysis at 37°C for 12 hours. Following enzymatic hydrolysis, liquid-liquid extraction was performed to extract the samples. Specifically, 2.5 mL of methyl tert-butyl ether and 0.5 mL of ethyl acetate were added to the centrifuge tube, followed by ultrasonication for 30 minutes and centrifugation at 3500 rpm for 15 minutes. After centrifugation, 2 mL of the upper organic phase was transferred to a plastic nitrogen blow-down tube, and the extraction process was repeated. During the second extraction, 3 mL of the upper organic phase was collected and transferred to the same plastic nitrogen blow-down tube. The plastic nitrogen blow-down tube was then placed in a nitrogen evaporator, and the organic phase was evaporated to dryness with a gentle stream of nitrogen gas. Once the sample was completely dried, the residue was reconstituted using 0.5 mL of methanol (ME). After reconstitution, the sample was filtered through a 0.22 μm membrane filter to remove potential particulate impurities or precipitates. Finally, the samples were labeled and stored at -20°C in a freezer.

An HPLC-MS/MS system equipped with a C18 column (1.7 μm, 2.1 mm i.d. × 50 mm length, ACQUITY UPLC BEH, Waters, Milford, MA, USA) will be used to analyze caffeine and creatinine samples (Waters XEVO G2-XS QTOF, UK). The system will operate in electrospray ionization positive mode (ESI) and use multiple reaction monitoring (MRM) mode for analysis.A 3.5 μL sample extract aliquot was injected. The mobile phase A (0.01% formic acid + 10 mM ammonium formate aqueous solution) and mobile phase B (acetonitrile) were gradient eluted. The flow rate was 0.400 mL/min, and the starting condition was 98% mobile phase A. Then, mobile phase B was ramped to 50% at 2 min and maintained at this level until 3 min. The gradient was then returned to the initial conditions and held at this level until 3.5 min. The electrospray ionization (ESI) source was operated with a desolvation temperature of 600° C and a capillary voltage of 2.5 kV to ensure efficient ionization of the analyte.
